# Therapeutic Effects of TN13 Peptide on Acute Respiratory Distress Syndrome and Sepsis Models In Vivo

**DOI:** 10.3390/jcm14061804

**Published:** 2025-03-07

**Authors:** Jae-Eun Byun, Jae-Won Lee, Eun Ji Choi, Juhyun Lee, Seok Han Yun, Chan Ho Park, Hanna Kim, Mi Sun Kim, Suk Ran Yoon, Tae-Don Kim, Ji-Yoon Noh, Sang-Hyun Min, Hyun-A. Seong, Kyung-Seop Ahn, Inpyo Choi, Haiyoung Jung

**Affiliations:** 1 Aging Convergence Research Center, Korea Research Institute of Bioscience and Biotechnology (KRIBB), Yuseong-gu, Daejeon 34141, Republic of Korea; quswodms@kribb.re.kr (J.-E.B.);; 2 Department of Biochemistry, School of Life Sciences, Chungbuk National University, Cheongju 28644, Republic of Korea; 3 Natural Medicine Research Center, Korea Research Institute of Bioscience and Biotechnology, Cheongju 28116, Republic of Korea; suc369@kribb.re.kr (J.-W.L.);; 4 Department of Biotechnology, University of Science and Technology (UST), Daejeon 34113, Republic of Korea; 5 Department of Functional Genomics, Korea University of Science and Technology (UST), Yuseong-gu, Daejeon 34113, Republic of Korea; 6 College of Pharmacy, Chungbuk National University, Cheongju 28160, Republic of Korea; 7 Immunotherapy Research Center, Korea Research Institute of Bioscience and Biotechnology (KRIBB), Yuseong-gu, Daejeon 34141, Republic of Korea; 8 Department of Innovative Pharmaceutical Sciences, Kyungpook National University, Daegu 41566, Republic of Korea; 9 Ingenium Therapeutics, 1662 Yuseong daero, Daejeon 34054, Republic of Korea

**Keywords:** TN13, p38 MAPK inhibitor, inflammation, ARDS, sepsis

## Abstract

**Background/Objectives:** Regulation of acute inflammatory responses is crucial for host mortality and morbidity induced by pathogens. The pathogenesis of acute respiratory distress syndrome (ARDS) and sepsis are associated with systemic inflammation. p38 MAPK is a crucial regulator of inflammatory responses and is a potential target for acute inflammatory diseases, including ARDS and sepsis. We investigated the therapeutic effects of the TAT-TN13 peptide (TN13) on severe inflammatory diseases, including ARDS and sepsis, in vivo. **Methods**: To establish the ARDS model, C57BL/6 mice were intranasally (i.n.) administered lipopolysaccharide (LPS; 5 mg/kg, 40 µL) to induce lung inflammation. As a positive control, dexamethasone (DEX; 0.2 mg/kg) was administered intraperitoneally (i.n.) 1 h post-LPS exposure. In the experimental groups, TN13 was administered intranasally (i.n.) at doses of 2.5 mg or 5 mg/kg at the same time point. In the LPS-induced sepsis model, mice received an intraperitoneal injection of LPS (20 mg/kg) to induce systemic inflammation. TN13 (25 mg/kg, i.p.) was administered 1 h after LPS treatment. Control mice received phosphate-buffered saline (PBS). Lung histopathology, inflammatory cell infiltration, cytokine levels, and survival rates were assessed to evaluate TN13 efficacy. **Results:** TN13 significantly reduced inflammatory cell recruitment and cytokine production in the lungs, thereby mitigating LPS-induced ARDS. In the sepsis model, TN13 treatment improved survival rates by suppressing inflammatory responses. Mechanistically, TN13 exerted its effects by inhibiting the p38 MAPK/NF-κB signaling pathway. **Conclusions**: These results collectively suggested that TN13 could be an effective treatment option for severe inflammatory diseases.

## 1. Introduction

Severe inflammatory responses induced by pathogens are critical for host mortality. Acute lung injury (ALI) and acute respiratory distress syndrome (ARDS) are representative of life-threatening lung injuries resulting in severe hypoxemia owing to an inflammatory response [1]. During the coronavirus disease 19 (COVID-19) pandemic, ARDS was a prominent cause of intensive care with respiratory failure [2,3]. Sepsis or septic shock is a severe inflammatory healthcare issue associated with infectious diseases that affects millions of patients each year. Excessive induction of inflammatory mediators from massive immune responses induces septic shock in patients. ARDS and sepsis have similar regulatory mechanisms, and severe sepsis is the most common etiology of ARDS [4]. Many reports suggest that sepsis-induced ARDS is more severe than ARDS caused by other factors and is the predominant cause of ARDS [5]. The mouse model of lipopolysaccharide (LPS)-induced lung injury is a well-characterized and commonly used model that mimics the pathophysiology of human ARDS. LPS is the most common inducer of sepsis models [6,7].

The pathogenesis of ARDS is governed by systemic inflammation, and an acute increase in circulating levels of pro-inflammatory cytokines, including IL-6, IL-1β, TNF-α, and interferon, induces the movement of immune cells into the site of infection. Some pro-inflammatory cytokines have been identified as biomarkers of ARDS [8,9]. p38 mitogen-activated protein kinase (MAPK) is the most critical kinase that regulates the production of pro-inflammatory cytokines and is reported to be a critical regulator of LPS-induced acute lung injury [10]. Inhibitors of p38 MAPK significantly reduced LPS-induced pulmonary inflammation [11]. The p38 MAPK family has four isoforms (α, β, γ, and δ), and the α isoform, especially, is more important than the others for the regulation of inflammation [12]. Many reports suggested that p38 MAPK is a potential target for the treatment of severe inflammatory diseases, including ARDS and sepsis. Thus, the development of p38 MAPK inhibitors might provide a more efficient therapeutic agent for acute inflammatory diseases in the future.

We had previously reported that thioredoxin-interacting protein (TXNIP) interacted with p38α MAPK and regulated its activity via docking interactions. We had developed a 13-amino acid peptide, which was derived from TXNIP and interacted with p38α MAPK. We conjugated a cell-penetrating peptide (CPP) derived from the human immunodeficiency virus (HIV) trans-activator protein (TAT) sequence (YGRKKRRQRRR) with the N-terminus of the 13-amino acid peptide (TN13) [13]. The peptide inhibited LPS-induced macrophage activation and significantly suppressed RANKL (receptor activator of nuclear factor-κB ligand)-mediated differentiation of osteoclasts [12,14].

In this study, we investigated the therapeutic effects of TN13 on severe inflammatory diseases, including ARDS and sepsis, in vivo. TN13 significantly improved survival in mouse models of ARDS and sepsis. We assessed inflammatory cell infiltration and inflammatory mediator levels in bronchoalveolar lavage fluid (BALF) following ARDS induction and found that TN13 effectively reduced inflammation. Additionally, TN13 markedly suppressed pro-inflammatory cytokine production and NF-κB transcriptional activation in LPS-induced sepsis. These results suggest that TN13 could be a potential treatment for severe inflammatory diseases.

## 2. Materials and Methods

### 2.1. Cell Culture and LPS Treatment

A549 cells were cultured in Roswell Park Memorial Institute 1640 (RPMI 1640) medium (Gibco, Grand Island, NY, USA), supplemented with 10% fetal bovine serum (FBS; R&D Systems, Minneapolis, MN, USA) and 1% antibiotics (Gibco, Grand Island, NY, USA). The cells were maintained at 37 °C in a humidified atmosphere with 5% CO_2_. For treatment, A549 cells were seeded at a density of 50,000 cells/cm^2^ in 60 mm plates. TN13 was pre-treated for 4 h at final concentrations of 10 µM and 20 µM before inducing inflammation. Following the pre-treatment, lipopolysaccharide (LPS; Sigma-Aldrich, St. Louis, MO, USA) was added to the medium at a final concentration of 2 µg/mL and incubated for 24 h to induce an inflammatory response. After LPS treatment, cells were harvested and analyzed for cytokine expression (IL-6, IL-1β, TNF-α) using ELISA kits (Invitrogen, Carlsbad, CA, USA), protein phosphorylation via Western blot analysis, and cell viability using the CCK-8 assay.

### 2.2. Cell Viability Assay

Cell viability was assessed using the Cell Counting Kit-8 (CCK-8; Dojindo, Kumamoto, Japan) following the manufacturer’s instructions. A549 cells were seeded in a 96-well plate at a density of 5 × 10^3^ cells/well and incubated overnight at 37 °C in a 5% CO_2_ atmosphere. The next day, cells were treated with LPS or TN13 for 24 h. After treatment, 10 µL of CCK-8 solution was added to each well and incubated for 2 h at 37 °C. The absorbance was measured at 450 nm using a microplate reader (BioTek, Winooksi, VT, USA). Cell viability was calculated as a percentage relative to the untreated control group.

### 2.3. Mouse Model of LPS-Induced ARDS and Sepsis

We used 8–12-week-aged wild-type male C57BL/6 mice, and they were purchased from KoaTech (Pyeongtaek, Republic of Korea). All mice were housed in a specific pathogen-free (SPF) animal facility under a 12 h light–dark cycle. Mice experiments were approved by the Institutional Animal Use and Care Committee of the Korea Research Institute of Bioscience and Biotechnology (approval no. KRIBB-AEC-24283) and were followed by the Guide for the Care and Use of Laboratory Animals published by the US National Institutes of Health. To induce the ARDS model, mice were intranasally (i.n.) administered LPS (5 mg/kg, 40 µL) to induce lung inflammation. One hour post-LPS exposure, mice were treated with either dexamethasone (DEX; 0.2 mg/kg, i.n.) or TN13 (2.5 mg/kg or 5 mg/kg, i.n.). Control mice received an equal volume of PBS (i.n.) instead of the treatment. For the LPS-induced sepsis model, mice were intraperitoneally (i.p.) administered LPS (20 mg/kg) to induce systemic inflammation. One hour post-LPS administration, TN13 (25 mg/kg, i.p.) was injected into the treatment group, while control mice were treated with an equal volume of PBS (i.p.).

### 2.4. TAT-TN13

A peptide derived from TXNIP, TAT-TN13 (Korean application, application number: 10-2017-0121017) (Peptron, Daejeon, Republic of Korea), TAT-cont: Y-G-R-K-K-R-R-Q-R-R-R, TAT-TN13: Y-G-R-K-K-R-R-Q-R-R-R-G-S-K-K-V-I-L-D-L-P-L-V-I. This peptide sequence binds to p38 MAPK, a representative kinase involved in inflammation, and specifically binds to p38α, one of the four p38 isomers. The synthesis and characteristics of this peptide have been reported in previous studies [13].

### 2.5. Lung Histology

Lung samples were fixed in 4% paraformaldehyde and were sliced at 5 µm thickness. Infiltration of inflammatory cells was determined using staining with hematoxylin and eosin (H&E) (Beyotime Institute of Biotechnology, Shanghai, China). We referred to the manufacturer’s instructions [15].

### 2.6. Cell Isolation and Flow Cytometry

Peripheral blood was collected from mice by securing them in a restrainer and performing tail vein sampling using a heparinized syringe to prevent coagulation. The collected blood was immediately diluted with phosphate-buffered saline (PBS) and processed for leukocyte isolation. Blood and spleen samples were filtered through a 70 μm cell strainer to obtain single-cell suspensions. Red blood cells were lysed using ACK lysis buffer (0.15 M NH_4_Cl, 1.0 mM KHCO_3_, 0.1 mM EDTA, pH 7.4) for 5 min at room temperature, followed by washing with cold PBS. For flow cytometry analysis, the isolated cells were resuspended in FACS buffer (PBS containing 2% fetal bovine serum) and incubated with the indicated antibodies for 20 min at 4 °C. Cells were then washed twice with cold PBS before analysis. The percentage and intensity of macrophage activation markers were assessed using the FACS Canto II flow cytometer (BD Biosciences, San Jose, CA, USA). The following antibodies were used: CD45.2 (cat. no. 558702; BD Biosciences, San Jose, CA, USA; APC), CD11B (cat. no. 552850; BD Biosciences, San Jose, CA, USA, PE-CY7), GR-1 (cat. no. 108418; BioLegend, San Diego, CA, USA; FITC), F4/80 (cat. no. 566787; BD, APC), CD80 (cat. no. 104714; BioLegend, San Diego, CA, USA; APC), and CD86 (cat. no. 105032; BioLegend, San Diego, CA, USA; Pacific Blue).

### 2.7. Bronchoalveolar Lavage Fluid (BALF) Collection and Analysis

BALF was collected as previously described [16]. To assess inflammatory responses, mice were anesthetized with Zoletil 50 (30–50 mg/kg, i.p.; Virbac, Carros, France) and xylazine (5–10 mg/kg, i.p.; Bayer Korea Ltd., Seoul, Republic of Korea) on day 3 post-experimentation. Tracheal PBS perfusion was performed 24 h after the final intraperitoneal treatment of MH and DEX, and BALF was collected from all the experimental groups. To evaluate inflammatory cell recruitment, BALF cells were mounted on glass slides, stained with Diff-Quik^®^ solution at room temperature for 30 s (Sysmex Corporation, Kobe, Japan), and manually counted under a light microscope (magnification, ×400). The levels of pro-inflammatory cytokines, including IL-1β, IL-6, and TNF-α, were quantified in BALF and mouse serum using ELISA kits (BD Biosciences, San Jose, CA, USA, Inc.; cat. nos. 558534, 555240, and 559603, respectively) and Duo-Set antibody pairs (R&D Systems, Minneapolis, MN, USA), following the manufacturer’s instructions. Cytokine absorbance was measured at 450 nm using a microplate multi-reader (SpectraMax i3x; Molecular Devices, San Jose, CA, USA).

### 2.8. Western Blotting and Antibodies

Following sample collection—including BALF, lung tissues, splenic cells, and RAW264.7 whole cells—samples were first washed twice with cold PBS. For protein extraction, tissues and cells were lysed using either RIPA I buffer (25 mM HEPES, pH 7.7; 0.3 M NaCl; 1.5 mM MgCl_2_; 0.2 mM EDTA; 0.1% Triton X-100; 10 mM β-glycerophosphate; 1 mM NaF; 1 mM Na_3_VO_4_) or CelLytic™ MT Cell Lysis Buffer (cat. no. c3228; Sigma-Aldrich; St. Louis, MO, USA) supplemented with appropriate protease (and phosphatase, when needed) inhibitor cocktails. Protein concentrations were determined using a BCA assay. Equal amounts of protein (50 µg per lane) were separated on 10–12% SDS-PAGE gels and transferred to PVDF membranes (Millipore, Sigma, Burlington, MA, USA). Membranes were blocked at room temperature for 1 h with 5% skim milk in 0.3% TBST. Subsequently, membranes were incubated overnight at 4°C with primary antibodies diluted in the blocking solution. The primary antibodies used were as follows: Anti-phosphorylated p38 (cat. no. 9211; Cell Signaling Technology, Inc., Danvers, MA, USA; 1:1000), Anti-p-IκBα (cat. no. 2859; Cell Signaling Technology, Inc., Danvers, MA, USA; 1:1000), Anti-phosphorylated p65 (cat. no. 3033; Cell Signaling Technology, Inc., Danvers, MA, USA; 1:1000), Anti-p65 (cat. no. 8242; Cell Signaling Technology, Inc., Danvers, MA, USA; 1:1000), Anti-β-actin (cat. no. sc-69879; Santa Cruz Biotechnology, Inc, Dallas, TX, USA.; 1:2000), Anti-p38 (cat. no. 9212; Cell Signaling Technology, Inc., Danvers, MA, USA; 1:1000), Anti-IκBα (cat. no. 15132; Invitrogen, Waltham, MA, USA; 1:1000). After primary incubation, membranes were washed with TBST at room temperature and then incubated for 1 h with HRP-conjugated secondary antibodies (goat anti-mouse and goat anti-rabbit; both 1:2000; cat. nos. 115-035-003 and 111-035-003, respectively; Jackson ImmunoResearch Laboratories, Inc., West Grove, PA, USA) at room temperature. Finally, immunoreactive bands were developed using Clarity™ Western ECL Substrate (cat. no. 170-5061; Bio-Rad Laboratories, Inc., Hercules, CA, USA) and visualized using either the WSE-6200 LuminoGraph II Imaging System (ATTO, Corporation, Tokyo, Japan) or an ImageQuant LAS 4000 mini luminescent Image Analyzer (Cytiva, Marlborough, MA, USA) Protein expression levels were quantified with ImageJ software (version 1.50e; National Institutes of Health, Bethesda, MD, USA), using β-actin as the loading control.

### 2.9. Quantitative Real-Time PCR and Primers

Total RNA was prepared using the RNeasy Mini Kit (Qiagen, Hilden, Germany), and cDNA was generated using a First-Strand cDNA synthesis kit (Toyobo, Osaka, Japan). Ten times diluted cDNA was subjected to qPCR using SYBR Premix ExTaq (Takara Bio, Shiga, Japan) and analyzed in the Applied Biosystems ViiA 7 Real-Time PCR System (Thermo Fisher Scientific, Waltham, MA, USA). The primers used were as follows: mouse GAPDH forward 5′-CTGCGACTTCAACAGCAACT-3′ and reverse 5′-GAGTTGGGATAGGGCCTCTC-3′, mouse TNF-α forward 5′-CAGGCGGTGCCTATGTCTCA-3′, and reverse 5′-GGCTACAGGCTTGTCACTCGAA-3′, mouse IL-1β 5′-TCCAGGATGAGGACATGAGCAC-3′, and reverse 5′-GAACGTCACACACCAGCAGGTTA-3′, mouse IL-6 5′-CAACGATGATGCACTTGCAGA-3′ and reverse 5′-CTCCAGGTAGCTATGGTACTCCAGA-3′, human GAPDH forward 5′-GCACCGTCAAGGCTGAGAAC-3′, and reverse 5′-TGGTGAAGACGCCAGTGGA-3′, human TNF-α forward 5′-TGCTTGTTCCTCAGCCTCTT-3′, and reverse 5′-CAGAGGGCTGATTAGAGAGA-3′, human IL-1β 5′-TCCAGGGACAAGGATATGGAG-3′, and reverse 5′-TCTTTCAACACGCAGGACAG-3′, human IL-6 5′-TACCCCCAGGAGAAGATTCC-3′, and reverse 5′-TTTTCTGCCAGTGCCTCTTT-3′.

### 2.10. Statistical Analysis

Results were expressed as the mean ± SD. *p*-values were analyzed using an unpaired *t*-test in the Prism program, and a value of *p* < 0.05 was considered statistically significant; * *p* < 0.05, ** *p* < 0.01, and *** *p* < 0.001 or # *p* < 0.05 vs. NC; * *p* < 0.05 vs. ARDS.

## 3. Results

### 3.1. TN13 Inhibited p38 MAPK/NF-κB Pathway Activation In Vitro

**Figure 1:**
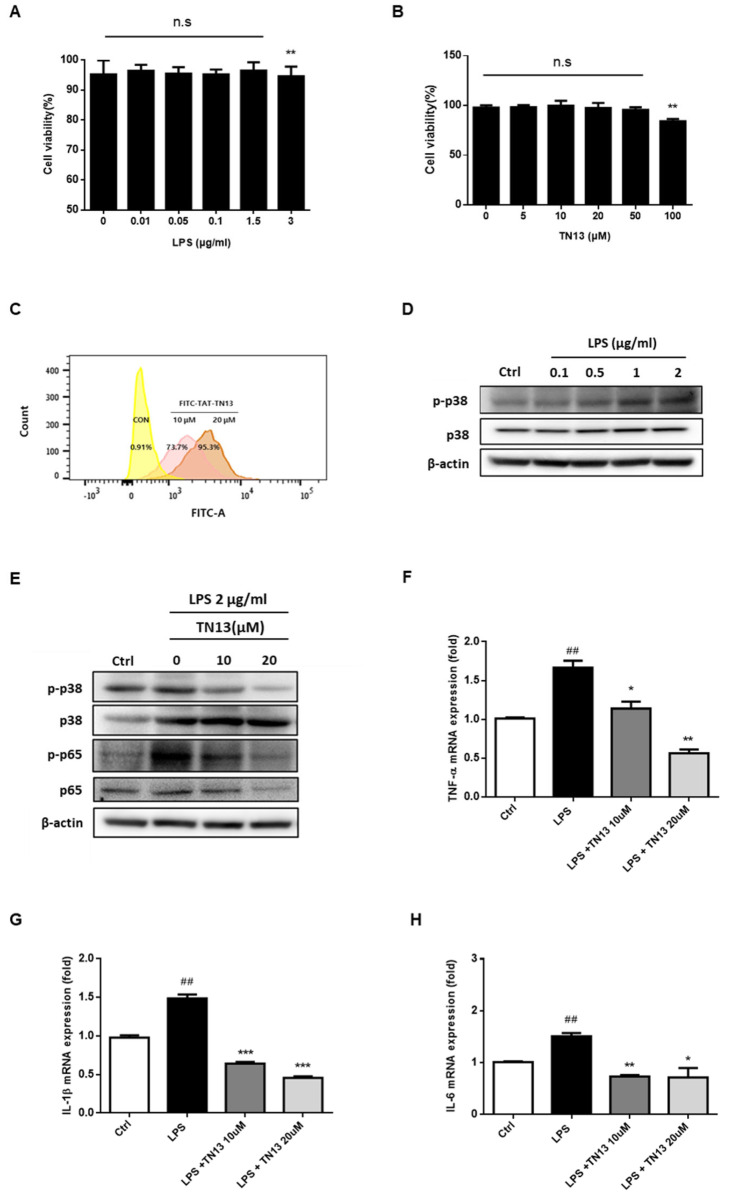
TN13 Peptide suppresses inflammation in A549 cells. (**A**,**B**) A549 cells were treated with various concentrations of LPS (**A**) or TN13 (**B**) for 24 h, and cell viability was assessed using the CCK-8 assay to evaluate cytotoxic effects. (**C**) Flow cytometry analysis was performed to examine the intracellular uptake of FITC-labeled TN13. (**D**) Western blot analysis was conducted to assess p38 MAPK phosphorylation following LPS treatment. (**E**) TN13 treatment was evaluated for its effect on LPS-induced phosphorylation of p38 MAPK. (**F**–**H**) The mRNA expression levels of key pro-inflammatory cytokines, including TNF-α (**F**), IL-1β (**G**), and IL-6 (**H**), were measured using quantitative real-time PCR. Data are presented as mean ± S.D. Statistical significance was determined using a two-tailed Student’s *t*-test, with ## *p* < 0.01 compared to the control group and * *p* < 0.05, ** *p* < 0.01, *** *p* < 0.001 compared to the LPS-treated group. n.s: indicates no statistical significance.

We first evaluated the anti-inflammatory effects of TN13 in A549 cells, a human alveolar epithelial cell line commonly used as an in vitro model for lung inflammation. These cells were selected due to their relevance in mimicking pulmonary inflammatory responses in ARDS [17]. Appropriate concentrations of LPS and TN13 were selected using cell viability assays (Figure 1A,B). TN13 was delivered to cells effectively in a dose-dependent manner within 4 h (Figure 1C). LPS-induced inflammatory responses were inhibited by TN13 treatment (Figure 1D,E), demonstrating that TN13 suppressed the p38 MAPK/NF-κB signaling pathways in A549 cells. Furthermore, the mRNA expression levels of pro-inflammatory cytokines, including TNF-α, IL-1β, and IL-6, were significantly reduced by TN13 treatment (Figure 1F–H). These findings indicate that TN13 effectively inhibits inflammatory signaling pathways in vitro, supporting its potential therapeutic role in lung inflammation.

### 3.2. Lung Injuries of Mice Against LPS-Induced ARDS Was Protected by TN13 Treatment

**Figure 2:**
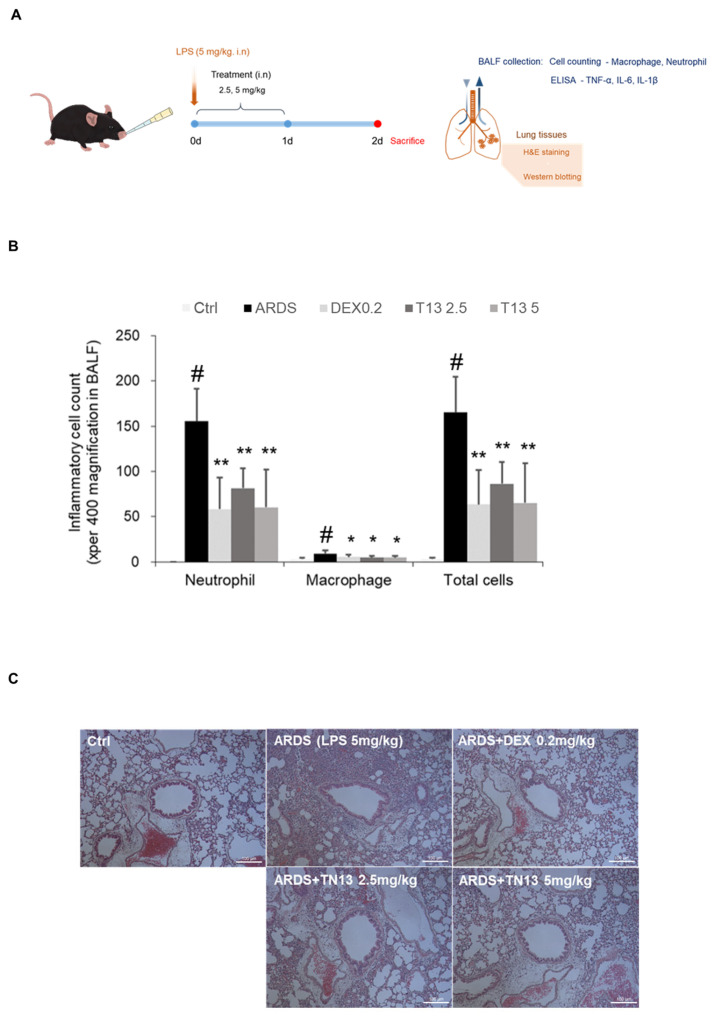
TN13 attenuates lung inflammation by reducing inflammatory cell infiltration in LPS-induced ARDS mice. (**A**) Experimental design and group composition: C57BL/6 mice (*n* = 5 per group) were randomly divided into the following five groups: Control: PBS only; LPS: ARDS induction with LPS (5 mg/kg, 40 µL, intranasal) only; Positive Control: LPS + dexamethasone (DEX, 0.2 mg/kg); Low-dose TN13: LPS + TN13 (2.5 mg/kg); and High-dose TN13: LPS + TN13 (5 mg/kg). LPS was administered on day 0, and TN13 or DEX was given intranasally 1 h post-LPS administration on days 0 and 1. Mice were sacrificed on day 2 for BALF collection and lung tissue harvesting. (**B**) Neutrophil and macrophage counts in BALF of mice were determined using Diff-Quik^®^ staining and cell counting (magnification, ×400; scale bar, 25 µM). (**C**) H&E staining in the lungs of mice (magnification, ×100; scale bar, 100 μm). Data are expressed as the mean ± SD. # *p* < 0.05 vs. Ctrl; * *p* < 0.05, ** *p* < 0.01 vs. ARDS.

**Figure 3:**
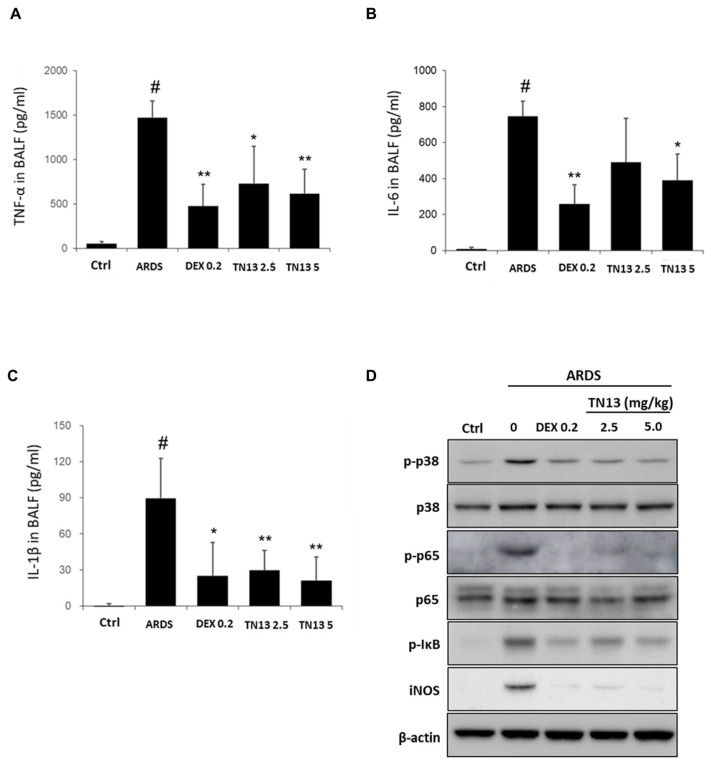
TN13 suppresses pro-inflammatory cytokine production in the lungs of LPS-induced ARDS mice. (**A**) TNF-α, (**B**) IL-6, and (**C**) IL-1β in the BALF of mice were determined using ELISA. (**D**) The p38/NF-κB pathway-related proteins were determined by Western blot in ARDS lungs. Data are expressed as the mean ± SD. # *p* < 0.05 vs. Ctrl; * *p* < 0.05,** *p* < 0.01 vs. ARDS.

To determine the efficacy of TN13 against acute inflammatory diseases, we established a mouse model of LPS-induced ARDS (Figure 2A). Inflammatory cell count and level of inflammatory cytokines in the BALF were determined as described previously [18]. Intranasal administration of LPS (5 mg/kg) remarkably induced acute inflammatory responses in mice. Intranasally administered TN13 (2.5 and 5 mg/kg) significantly inhibited the increase in neutrophil and macrophage numbers in BALF in a dose-dependent manner, and it was comparable to that of 0.2 mg/kg DEX, which was used as a positive control (Figure 2B). The levels of TNF-α (Figure 3A), IL-6 (Figure 3B), and IL-1β (Figure 3C) were also decreased by TN13 treatment. The activation of p38 MAPK and NF-κB signaling by LPS treatment had been well characterized in the lungs during severe inflammatory responses [18]. TN13 treatment significantly suppressed LPS-induced p38 MAPK and NF-κB activation in the lungs of mice (Figure 3D). In addition, H&E staining of the lungs showed the infiltration of inflammatory cells around the airway. DEX and TN13 treatment remarkably reduced the recruitment of inflammatory cells around the airway in the lungs (Figure 2C). These results suggested that TN13 exerted protective effects against LPS-induced ARDS by inhibiting inflammatory cell recruitment and cytokine production.

### 3.3. TN13 Treatment Rescued Mice Against LPS-Induced Sepsis

**Figure 4:**
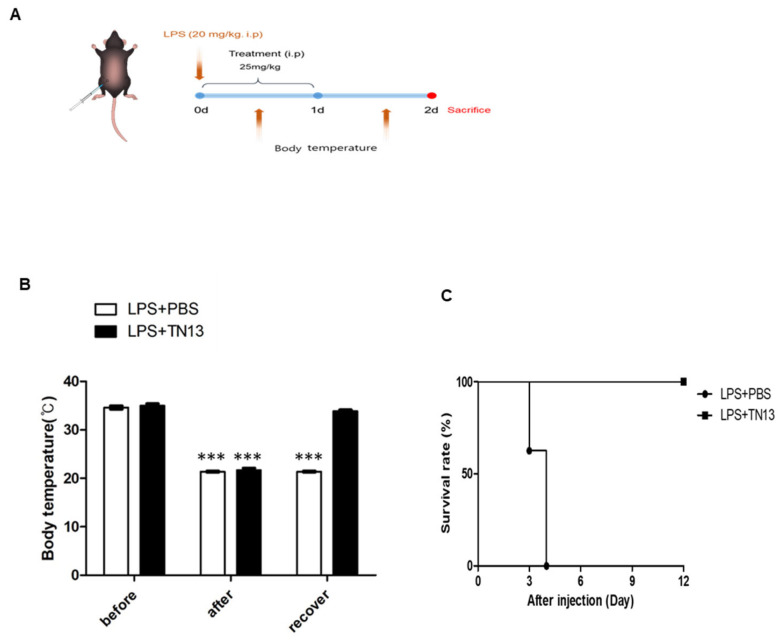
TN13 rescues mice from LPS-induced sepsis. (**A**) Experimental design of sepsis mouse model. Schematic illustration for routine i.p. of LPS stimulation (20 mg/kg) and treatment of TN13 (25 mg/kg) injection once a day a total of 2 times. (**B**) Mice body temperature change after LPS + PBS or LPS + TN13 injection (*n* = 10). Before, −1 h; after, +1 h; and recover, +24 h after TN13 (25 mg/kg) treatment, which was administered 1 h after LPS injection. (**C**) Mice survival over time LPS + PBS or LPS + TN13 injection (*n* = 10). Data are expressed as the mean ± SD. *** *p* < 0.001 vs. before control.

To investigate the function of TN13 on another acute inflammatory disease, we used a mouse model of LPS-induced sepsis. We intraperitoneally administered LPS (20 mg/kg) to the mice that died in five days after sepsis induction. TN13 (25 mg/kg) was intraperitoneally injected twice into the mice (Figure 4A) and efficiently rescued the low body temperature and increased the survival of mice with LPS-induced sepsis (Figure 4B,C). These results suggested that TN13 could rescue mice from severe sepsis.

### 3.4. Inflammatory Responses in LPS-Induced Sepsis Was Suppressed by TN13 Treatment

**Figure 5:**
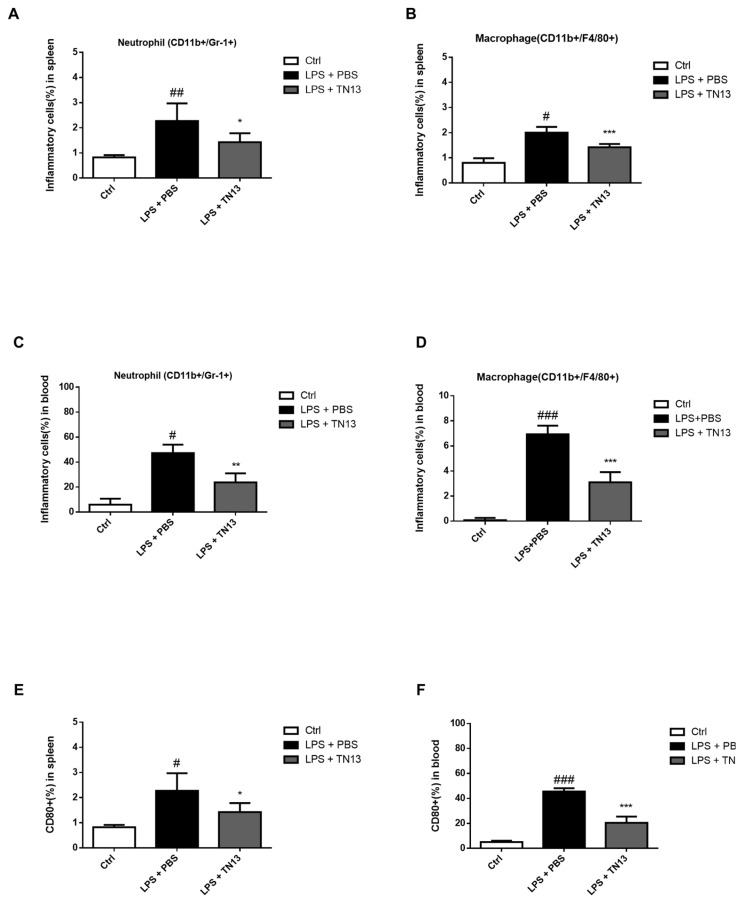
TN13 inhibits inflammatory responses in the sepsis mouse model. (**A**–**D**) Frequencies of neutrophil and macrophage in the spleen and peripheral blood of mice (*n* = 5). (**E**,**F**) Frequencies of activated macrophages (CD80+ cells) in the spleen and peripheral blood. Cells were analyzed by flow cytometry to determine their percentage. Data are mean ± S.D. (Statistical significance was determined using a two-tailed Student’s *t*-tests. # *p* < 0.05, ## *p* < 0.01, ### *p* < 0.001 vs. Ctrl; * *p* < 0.05, ** *p* < 0.01, *** *p* < 0.001 vs. LPS + PBS).

**Figure 6:**
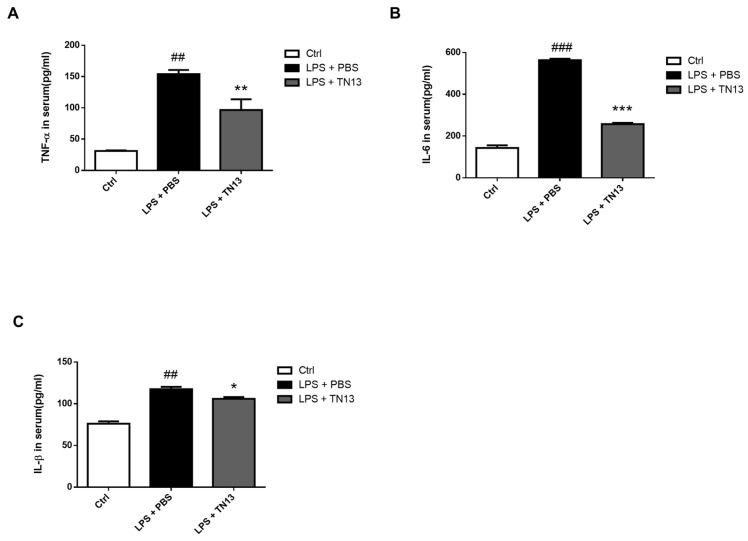
TN13 downregulates inflammatory cytokine levels in lipopolysaccharide-induced sepsis mouse model. Secretions of cytokine TNF-α (**A**), IL-6 (**B**), and IL-1β (**C**) were determined in mice serum using ELISA (*n* = 10). Data are mean ± S.D. (Statistical significance was determined using a two-tailed Student’s *t*-tests. ## *p* < 0.01, ### *p* < 0.001 vs. Ctrl; * *p* < 0.05,** *p* < 0.01, *** *p* < 0.001 vs. LPS + PBS).

As shown in the mouse model of ARDS, TN13 remarkably rescued the mice from severe sepsis. To address the immunological effects of TN13 in the severe sepsis model, we analyzed the distribution of inflammatory cells in the spleen and blood. TN13 treatment significantly decreased the frequencies of neutrophil and macrophage in the spleen and blood (Figure 5A–D) and also reduced macrophage activation (Figure 5E,F). In addition, TN13 treatment suppressed the induction of pro-inflammatory cytokines in the serum of mice (Figure 6A–C). These results collectively demonstrated that TN13 inhibited inflammatory responses in a mouse model of LPS-induced sepsis.

### 3.5. p38 MAPK/NF-κB Activation Was Inhibited by TN13 Treatment

**Figure 7:**
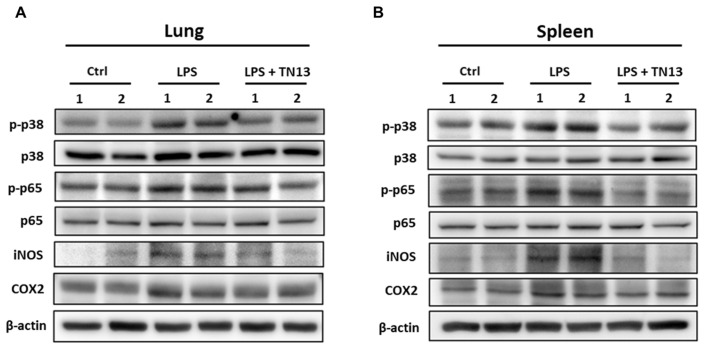
TN13 inhibits the p38/NF-κB pathways in the sepsis mouse model. The expressions of the p38/NF-κB pathway-related protein were determined by Western blot in sepsis lung (**A**) and spleen (**B**).

The activation of the p38 MAPK/NF-κB signaling pathway is prominent in acute inflammatory diseases, as found in the lungs and spleens of mice with LPS-induced sepsis. TN13 treatment significantly reversed the activation of the p38 MAPK/NF-κB signaling pathway caused by LPS treatment in the lungs and spleens (Figure 7A,B). The results indicated that TN13 could inhibit severe sepsis by inhibiting the p38 MAPK/NF-κB signaling pathway.

## 4. Discussion

p38 MAPK is a key regulator of inflammatory responses in both in vitro and in vivo models, making it a promising therapeutic target for acute inflammatory diseases such as ARDS and sepsis. However, despite extensive research, clinical trials of p38 MAPK inhibitors have shown limited success due to dose-limiting toxicity, poor specificity, and off-target effects [12,13]. Therefore, identifying novel inhibitors with improved safety and efficacy profiles remains a critical challenge.

In our previous studies, we developed TN13, a soluble peptide inhibitor that selectively targets p38α MAPK [12,13]. Unlike conventional small-molecule inhibitors, TN13 exhibits high specificity and fewer off-target effects, making it a promising candidate for treating inflammatory diseases. Previous studies have shown that inhibiting TXNIP expression leads to significant reductions in lung inflammation and cytokine production in ALI models [19,20]. Our findings further support this by demonstrating that TN13, which targets the TXNIP-p38 MAPK axis, effectively suppresses inflammatory responses and improves survival in sepsis and ARDS models. These results highlight the potential of TXNIP modulation as a therapeutic approach for acute inflammatory diseases. Conventional p38 MAPK inhibitors have limited clinical applications due to poor specificity, toxicity, and off-target effects. TN13, a peptide-based inhibitor derived from TXNIP, specifically targets p38α MAPK.

This study is the first to demonstrate the in vivo therapeutic effects of TN13 in ARDS and sepsis models, highlighting its anti-inflammatory properties via p38 MAPK/NF-κB pathway regulation. Expanding on previous in vitro studies, we show that TN13 has systemic effects and improves survival rates. With greater specificity and fewer side effects than conventional inhibitors, TN13 emerges as a promising therapeutic candidate for severe inflammatory diseases. Our earlier research demonstrated that TN13 inhibits LPS-induced macrophage activation and significantly suppresses RANKL-mediated osteoclast differentiation [12,14]. Based on these findings, we hypothesized that TN13 could serve as an effective therapeutic agent against acute inflammatory diseases mediated by p38 MAPK activation. To test this hypothesis, we evaluated the therapeutic effects of TN13 in two murine models of acute inflammation: LPS-induced ARDS and sepsis. Our results revealed that TN13 effectively inhibited inflammatory cell recruitment and reduced cytokine production, thereby mitigating lung injury in ARDS. Notably, in the LPS-induced sepsis model, TN13 treatment significantly improved survival rates, with all treated mice surviving (Figure 4C). These findings underscore the potential of TN13 as a life-saving treatment for systemic inflammation.

To further validate TN13 anti-inflammatory effects, we performed in vitro experiments using A549 cells, a well-established human alveolar epithelial cell line widely used to study lung inflammation. Our CCK-8 cell viability assay (Figure 1A,B) confirmed that TN13 did not induce cytotoxicity at therapeutic concentrations, ensuring its suitability for clinical translation. Additionally, we observed that TN13 effectively suppressed LPS-induced inflammatory responses in A549 cells, including the expression of key pro-inflammatory cytokines such as IL-6, TNF-α, and IL-1β. These findings strongly correlate with our in vivo results, reinforcing the conclusion that TN13 mitigates lung inflammation by targeting the p38 MAPK/NF-κB signaling pathway.

The COVID-19 pandemic has highlighted the urgent need for effective treatments against virus-induced ARDS and sepsis, which remain major causes of mortality in critically ill patients. Given that SARS-CoV-2 infection directly activates the p38 MAPK pathway, many researchers have suggested that p38 MAPK inhibitors could serve as potential treatments for COVID-19-induced hyper inflammation. Recent studies have shown that inhibiting p38 MAPK reduces SARS-CoV-2 replication and suppresses the inflammatory response. Since TN13 selectively targets p38α MAPK, it may offer a dual benefit by both inhibiting viral replication and controlling excessive inflammation. Although we did not directly investigate TN13′s effects on COVID-19-induced ARDS and sepsis, our findings provide a strong rationale for future research in this area. Future studies should evaluate the therapeutic potential of TN13 in viral infection models, particularly in the context of COVID-19 or other viral pneumonia-related ARDS. Despite the promising findings of this study, several limitations should be considered. First, this study utilized an LPS-induced ARDS and sepsis model, which, while well established for studying inflammatory responses, does not fully reflect the complex pathophysiology of human ARDS and sepsis. Further studies should incorporate clinically relevant models that better mimic the disease’s pathological progression. However, it is important to note that TN13’s potential therapeutic effects were clearly demonstrated in this study, strongly supporting its anti-inflammatory role in sepsis and ARDS models. Given its efficacy in reducing inflammatory responses, TN13 may have clinical applicability in treating hyper inflammatory conditions, bringing it one step closer to potential clinical translation. Additionally, while TN13 demonstrated efficacy in reducing inflammation, its long-term safety profile and pharmacokinetics were not assessed in this study. We should evaluate potential toxicity and optimal dosing strategies to ensure its feasibility for clinical applications.

Lastly, this study primarily focused on the p38 MAPK/NF-κB pathway, but other inflammatory signaling pathways may also play an important role in ARDS and sepsis. Further mechanistic studies are needed to comprehensively understand TN13’s therapeutic potential and its broader impact on inflammatory diseases. In summary, we demonstrated that TN13 effectively protects against LPS-induced ARDS and sepsis by suppressing inflammatory responses via the p38 MAPK/NF-κB pathway. Compared to conventional p38 MAPK inhibitors, TN13 exhibits greater specificity, reduced toxicity, and promising therapeutic potential. Based on these findings, TN13 represents a novel and promising treatment strategy for severe inflammatory diseases, including ARDS and sepsis. Further research should focus on its translational potential in clinical settings and viral infection-induced inflammatory diseases.

## 5. Conclusions

In this study, we demonstrated that TN13 effectively inhibits the p38 MAPK/NF-κB signaling pathway, thereby suppressing inflammatory responses both in vitro and in vivo. In vitro, TN13 significantly reduced inflammatory cytokine production in A549 cells, confirming its direct anti-inflammatory effects at the cellular level. In vivo, TN13 administration reduced inflammatory cell infiltration in lung tissues, modulated systemic immune responses, and improved survival in LPS-induced ARDS and sepsis models. These findings highlight TN13’s strong therapeutic potential for acute inflammatory diseases.

Given the critical role of p38 MAPK in viral infections, TN13 may also provide therapeutic benefits in virus-induced hyper inflammation, including COVID-19-related ARDS and sepsis. Future studies will focus on evaluating its efficacy in viral infection models to further explore its clinical applicability. By expanding upon these findings, TN13 could serve as a promising candidate for clinical translation in the treatment of ARDS, sepsis, and other inflammatory conditions.

## Data Availability

The data generated in the present study may be requested from the corresponding author.

## Abbreviations

ARDS	Acute respiratory distress syndrome
LPS	Lipopolysaccharide
PBS	Phosphate-buffered saline
i.p.	Intraperitoneally
i.n.	Intranasally
DEX	Dexamethasone
ALI	Acute lung injury
COVID-19	Coronavirus disease 19
p38 MAPK	p38 mitogen-activated protein kinase
NF-κB	Nuclear factor kappa-light chain enhancer of activated B
TXNIP	Thioredoxin-interacting protein
CPP	Cell-penetrating peptide
HIV	Human immunodeficiency virus
TAT-TN13	Trans-activator protein-TN13
RANKL	Receptor activator of nuclear factor-κB ligand
BALF	Bronchoalveolar lavage fluid
